# Instrumentalisation dangereuse du risque d’épidémie de maladie à virus Ebola à Mayotte

**DOI:** 10.48327/mtsi.v6i3.2026.882

**Published:** 2026-08-17

**Authors:** Pierre GAZIN

**Affiliations:** SFMTSI, Institut Pasteur, 25 rue du Dr Roux, 75015 Paris, France

**Figure:**
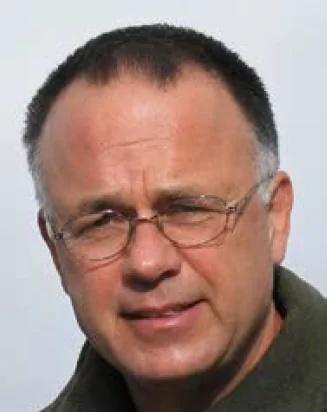

L’île de Mayotte est un département français situé dans le nord du canal du Mozambique à 350 km de Madagascar, à 520 km de la côte africaine la plus proche et à 70 km d’Anjouan, île appartenant aux Comores. De taille réduite (375 km^²^) et fortement peuplé (environ 330 000 habitants, densité de 880 habitants/km^²^), ce territoire est soumis à une forte immigration non contrôlée depuis les Comores voisines et quoiqu’à un niveau beaucoup plus faible depuis le continent africain, en particulier depuis l’est de la République démocratique du Congo (RdC). Cette situation crée de fortes tensions entre la population mahoraise et les migrants.

Une nouvelle épidémie de maladie d’Ebola (MVE) due au virus Bundibugyo a été déclarée par la RdC. La zone la plus atteinte est la province d’Ituri, à l’est du pays, frontalière avec l’Ouganda. Les premiers cas sont probablement apparus en mars 2026. L’insécurité et l’instabilité de la région du fait de mouvements armés n’ont pas permis de confirmer l’épidémie avant le mois de mai [4]. Au 4 août 2026, 3 802 cas confirmés ont été déclarés en RdC avec 1 707 décès. Parmi ces cas, 3 317 ont été observés en Ituri, 414 au Nord-Kivu et 3 au Sud-Kivu. En Ouganda, 20 cas ont été notifiés dont 2 décès, la dernière notification datant du 20 juin. Quinze de ces cas étaient en rapport avec des déplacements depuis la RdC. L’épidémie est considérée comme terminée en Ouganda depuis le 28 juillet 2026 [2]. Le taux de létalité de l’ensemble des cas confirmés atteint 45 %.

Le 20 mai 2026, une députée de Mayotte a posé une question orale au gouvernement sur le risque d’introduction de la MVE à Mayotte : « …Mayotte est en état d’urgence migratoire permanent. Notre frontière est, vous le savez, une passoire. Chaque jour quasiment, les *kwassas* (pirogues motorisées adaptées à la navigation en mer) débarquent des dizaines de clandestins sur nos plages : des Comoriens, des Africains qui arrivent, entre autres, de RdC, pays frappé par le virus d’Ebola… Ces migrants, entrés illégalement, sont certainement déjà allés rejoindre le camp sauvage de Tsoundzou, qui rassemble des milliers d’Africains installés dans la mangrove, dans l’indignité la plus abjecte … » [3].

Ce camp spontané de Tsoundzou, situé en bord de mer, regroupe environ 1 400 migrants majoritairement provenant de RdC. Les conditions de vie y sont très précaires. La densité de l’habitat fait de bambous et bâches est extrême. L’arrivée d’un cas de porteur de virus Bundibugyo pourrait effectivement être catastrophique.

Cette introduction du virus est-elle possible ?

Après une incubation de 2 à 21 jours, 7 jours en moyenne, la MVE se manifeste par une attaque fébrile et une atteinte très marquée de l’état général. Avant les signes cliniques, le sujet infecté n’est pas contagieux. Il devient très contagieux quand la maladie est déclarée et jusqu’à sa guérison ou son décès.

Depuis l’est de la RdC, les routes migratoires passent par l’Ouganda et la Tanzanie, soit environ 1 700 km pour atteindre le littoral puis, par bateau, 800 km jusqu’aux abords d’Anjouan et, enfin, plusieurs heures de pirogue pour arriver à Mayotte. Un individu atteint par la MVE n’est pas en capacité de voyager. L’introduction du virus ne pourrait se faire qu’à partir d’un porteur sain ayant fait rapidement ce trajet, moins de 21 jours, et déclarant la maladie seulement après son arrivée. La probabilité de cet événement n’est pas nulle mais elle est très faible.

Un aspect positif est que les migrants ont une relation de confiance avec les divers acteurs humanitaires agissant dans le camp de Tsoundzou et qu’ils ont intérêt à se déclarer auprès des autorités administratives. La possibilité d’asile politique existe pour un habitant de la RdC ayant fui la violence endémique de cette partie du pays et cela est connu.

Il est ainsi regrettable qu’un député entretienne les peurs autour des risques infectieux, surtout après l’avoir déjà fait en février 2026 pour le Mpox [1]. Au-delà d’une inutile stigmatisation des migrants originaires de la région des Grands Lacs, il en résulte un risque non négligeable de violences irrationnelles entre communautés.
