# Supplementary material for: Dangerous exploitation of the risk of an Ebola virus disease outbreak in Mayotte
Source: Med Trop Sante Int. 2026 Aug 17;6(3):mtsi.v6i3.2026.882. [Article in French] doi: 10.48327/mtsi.v6i3.2026.882 (PMC13697901; doi:10.48327/mtsi.v6i3.2026.882)
Supplement: Related texts

# Research article: Dangerous exploitation of the risk of an Ebola virus disease outbreak in Mayotte

Mayotte, a French department, is located in the northern part of the Mozambique Channel. It is 350 km away from Madagascar, 520 km from the nearest African coast, and 70 km from Anjouan, an island belonging to the Comoros. This small territory (375 km^²^) is densely populated (with approximately 330,000 inhabitants and a population density of 880 people per km^²^). It is subject to high levels of uncontrolled immigration from the neighboring Comoros Islands and, to a much lesser extent, from the African mainland, particularly from eastern Democratic Republic of the Congo (DRC). This situation has created significant tensions between the local Mayotte population and the migrants.

The DRC has declared a new Ebola virus disease (EVD) outbreak caused by the Bundibugyo virus. Ituri Province, in the eastern part of the country bordering Uganda, has been hit hardest. The first cases likely appeared in March 2026. Insecurity and instability in the region due to armed groups prevented confirmation of the outbreak until May [4]. As of August 4, 2026, the DRC had reported 3,802 confirmed cases, resulting in 1,707 deaths. Of these, 3,317 were in Ituri, 414 were in North Kivu, and three were in South Kivu. In Uganda, 20 cases were reported, including two deaths. The most recent report was on June 20. Fifteen of these cases were linked to travel from the DRC. The outbreak in Uganda was considered over as of July 28, 2026 [2]. The case fatality rate for all confirmed cases is 45%.

On May 20, 2026, a National Assembly member from Mayotte asked the government about the risk of introducing EVD to Mayotte. “Mayotte is in a permanent state of migration emergency. As you know, our border is a sieve. Almost every day, the *kwassas* (motorized canoes designed for ocean navigation) unload dozens of illegal immigrants on our beaches. Comorians and Africans arriving from countries such as the DRC, which is affected by the Ebola virus… These illegal immigrants have likely already arrived at the makeshift camp in Tsoundzou, where thousands of Africans live in abject conditions in the mangrove swamp…” [3].

The camp is located by the sea and is home to approximately 1,400 migrants, most of whom come from the DRC. Living conditions there are extremely precarious. The shelters, made of bamboo and tarps, are extremely dense. The arrival of someone carrying the Bundibugyo virus could be catastrophic indeed.

Could the virus be introduced in this way?

After an incubation period of two to 21 days—seven days on average—Ebola virus disease (EVD) manifests as a fever and severe deterioration in health. Before clinical symptoms appear, the infected individual is not contagious. They become highly contagious once the disease manifests and remain so until they recover or die.

Migration routes from eastern DRC pass through Uganda and Tanzania—a distance of approximately 1,700 km—to reach the coast. From there, the journey continues by boat for 800 km to the vicinity of Anjouan. Finally, it takes several hours by canoe to reach Mayotte. An individual infected with MVE would be unable to make this journey. The virus could only be introduced by an asymptomatic carrier who could make this journey in less than 21 days and develop symptoms only after arrival. While the probability is not zero, it is very low.

One positive aspect is that migrants trust the various humanitarian organizations operating in the Tsoundzou camp. Registering with the administrative authorities is in their best interest. A resident of the DRC who has fled the endemic violence in that part of the country can apply for political asylum, and this is well known.

Therefore, it is regrettable that a member of parliament is fueling fears about infectious risks, especially after doing so in February 2026 regarding Mpox [1]. Beyond the unnecessary stigmatization of migrants from the Great Lakes region, this creates a significant risk of intercommunity violence.
